# Radiosensitization by Marine Sponge *Agelas* sp. Extracts in Hepatocellular Carcinoma Cells with Autophagy Induction

**DOI:** 10.1038/s41598-018-24745-w

**Published:** 2018-04-20

**Authors:** Changhoon Choi, Arang Son, Hyi-Seung Lee, Yeon-Ju Lee, Hee Chul Park

**Affiliations:** 1 Department of Radiation Oncology, Samsung Medical Center, Seoul, 06351 South Korea; 2 Marine Natural Product Chemistry Laboratory, Korea Institute of Ocean Science, and Technology, Ansan, 426-744 South Korea; 3 Sungkyunkwan University School of Medicine, Seoul, 06351 South Korea

## Abstract

Although radiation therapy is an effective treatment modality in many cancers, there is an urgent need to develop therapeutic drugs capable of overcoming radioresistance or minimizing normal tissue toxicity. A wide variety of marine-derived bioactive compounds have been screened for anti-cancer drug discovery, but little is known regarding radiation therapy applications. In this study, six different extracts of marine sponges collected from the Micronesian sea were screened for anti-cancer and radiosensitizing activity. Two extracts derived from *Agelas* sponges collected off the coast of Kosrae and Chuuk, the Federated States of Micronesia significantly decreased clonogenic survival of hepatocellular carcinoma (HCC) cells after exposure to ionizing radiation (IR). The *Agelas* extracts augmented IR-induced apoptosis and accumulation of reactive oxygen species (ROS). Endoplasmic reticulum (ER) stress was increased via unfolded protein response stimulation, which induced autophagy. N-acetylcysteine, a ROS scavenger, diminished ER stress and autophagy induction effects. This result indicated that *Agelas* extracts may sensitize HCC cells to IR via ROS overproduction *in vitro*. Our findings suggest that the *Agelas* sp. may have potential utility in radiosensitizer development.

## Introduction

Cancer is a leading cause of death worldwide and development of anticancer drugs with improved sensitivity and specificity is urgently needed. Most anticancer drugs used clinically are based on chemically synthesized compounds, but bioactive molecules from natural organisms have been intensively investigated as novel drug sources. Bioactive compounds from marine organisms have received increased attention due to their diversity and complex habitats^1,2^. Bioactive molecules include proteins, peptides, amino acids, fatty acids, polysaccharides, phenolic compounds, photosynthetic pigments, and vitamins. A variety of marine bioactive molecules have been tested as candidates for lead compounds in anticancer drug discovery^3–6^.

Radiation therapy is a powerful treatment modality that is applied for approximately 50% of cancer patients. Recent advances in beam delivery techniques have led to an improvement of treatment outcome with better tumour control and minimal injury to normal tissues. Nonetheless, treating radiation resistance tumours is challenging due to tumour recurrence^7,8^. Much effort has been devoted to developing radiosensitizers, chemical compounds that potentiate the anti-cancer effect of radiation therapy, to increase local tumour control^9–11^. Major modes of radiation-induced tumour cell death include apoptosis, necrosis, and autophagy, which are mainly caused by accumulation of unrepairable DNA breaks^12^. Various chemotherapy drugs that possess DNA-damaging activity have been shown to exert a radiation-sensitizing effect, but they accompanied by significant adverse effects. Hypoxia-targeted agents are another mainstay of radiosensitizers that alleviate resistance to radiation therapy^9^. Molecular-targeted agents have also been shown to provide a synergistic effect with radiation therapy^11^.

Hepatocellular carcinoma (HCC), a primary malignancy of the liver, is the fifth most common cancer type and third most common cause of cancer-related death worldwide. Radiation therapy is recommended in combination with transarterial chemoembolization (TACE) for patients with advanced HCC who are not amenable to curative therapy options such as surgery, transplantation or radiofrequency ablation^13^. Sorafenib is a multi-targeted kinase inhibitor and the only Food and Drug Administration (FDA)-approved drug for the advanced HCC, but its efficacy is limited. Comprehensive genomic studies have provided clues for molecular targeted therapeutics or new treatment strategies, but little success has been reported^14^.

Investigations of bioactive compounds from terrestrial plants have demonstrated that compounds such as curcumin and quercetin could have potential as natural radiosensitizers^15^. However, applications of marine bioactive compounds have not been reported regarding radiation therapy. In this study, we obtained the extracts of marine species collected from the Micronesian ocean and evaluated the HCC cytotoxicity and radiation sensitization activity. We reported for the first time that marine sponges of the *Agelas* sp. may be a source of natural compounds with radiosensitizing activity.

## Results

### A blind screen of anticancer and radiosensitizing activities in marine sponge extracts

**Figure 1:**
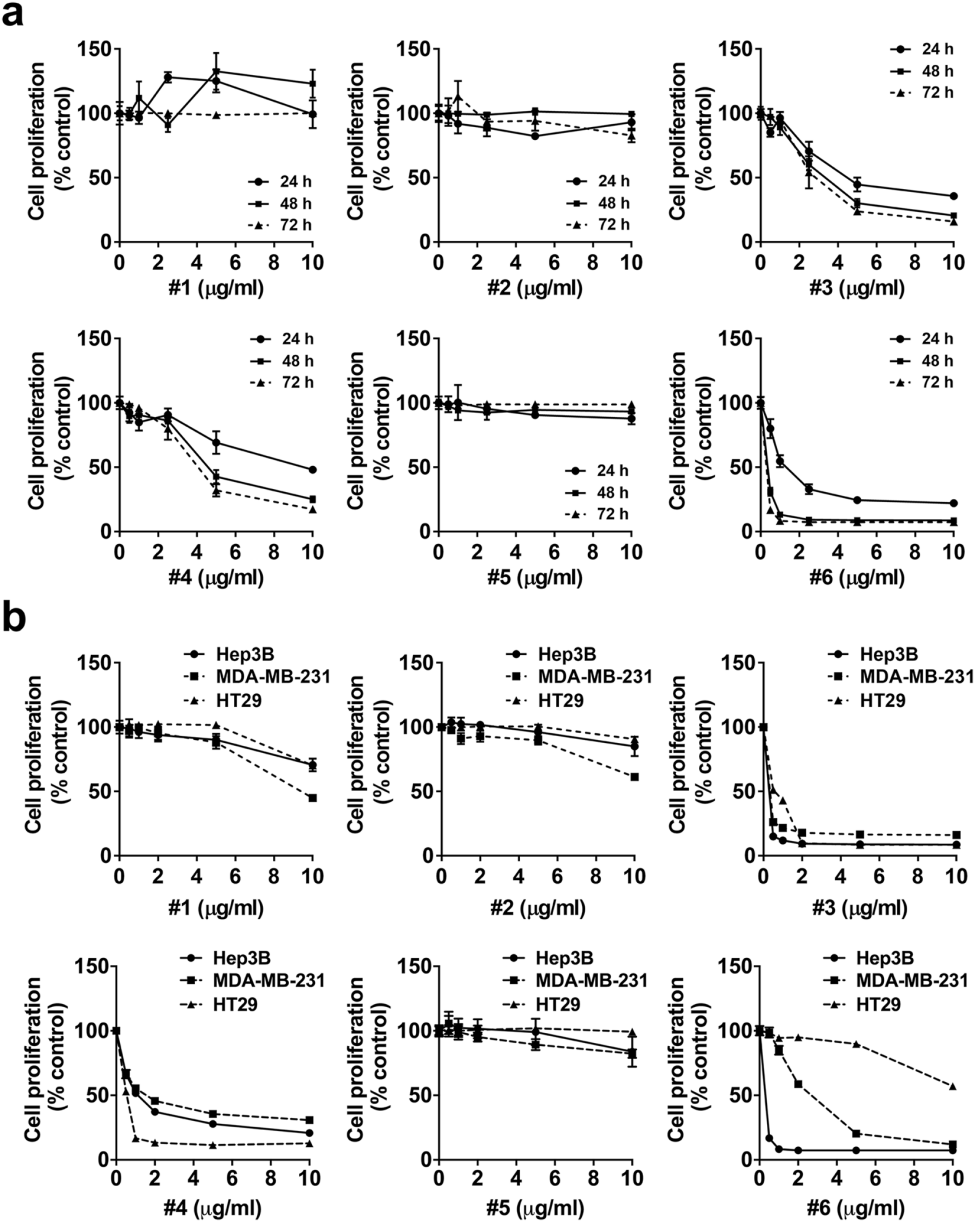
Screen of anticancer and radiosensitizing activity in the extracts of marine sponges collected from the Micronesian ocean. (**a**) Cytotoxicity of six marine sponge extracts were screened in human hepatocellular carcinoma Hep3B cells. Hep3B cells were incubated with either DMSO or the indicated concentrations of marine sponge extracts for 24, 48 and 72 h. Cytotoxicity was determined using a CCK-8 assay kit. Three of the samples had severe cytotoxicity in Hep3B cells. (**b**) Cytotoxicity of the six marine sponge extracts were compared in Hep3B, breast cancer MDA-MB-231, and colorectal cancer HT29 cells. The cells were incubated with the indicated concentrations of the sponge extracts for 72 h.

We selected six methanol extracts of marine sponges collected from the Micronesian ocean and identified marine-derived bioactivity with potential application for radiation therapy. We measured the time and concentration-dependent cytotoxicity of the extracts in human HCC Hep3B cells. A blind screen revealed that three of six samples (#3, #4, and #6) had a strong cytotoxicity effect in Hep3B cells, but the rest (#1, #2, and #5) had minimal effects on cell proliferation even at a concentration of 10 µg/mL (Fig. 1a). The cytotoxic extracts were prepared from *Theonella* sp. (#3 and #4) and *Haliclona* sp. (#6). Extracts with mild cytotoxic activity were prepared from *Agelas* sp. (#1 and #2) and *Coscinoderma* sp. (#5). Similar cytotoxic profiles were observed in other cancer types, including breast cancer MDA-MB-231 cells and colorectal cancer HT29 cells, except that #6 showed mild cytotoxicity against HT29 cells (Fig. 1b). The sponge extracts used in this study are summarized in Table 1.

**Table 1:** Marine sponge extracts used in this study.

No	Sponge species	State	Country
1	*Agelas* sp.	Chuuk State	Federated States of Micronesia
2	*Agelas* sp.	Kosrae state	Federated States of Micronesia
3	*Theonella* sp.		Philippines
4	*Theonella* sp.		Philippines
5	*Coscinoderma* sp.	Kosrae state	Federated States of Micronesia
6	*Haliclona* sp.		Philippines

### The *Agelas* extracts have radiosensitizing activity

**Figure 2:**
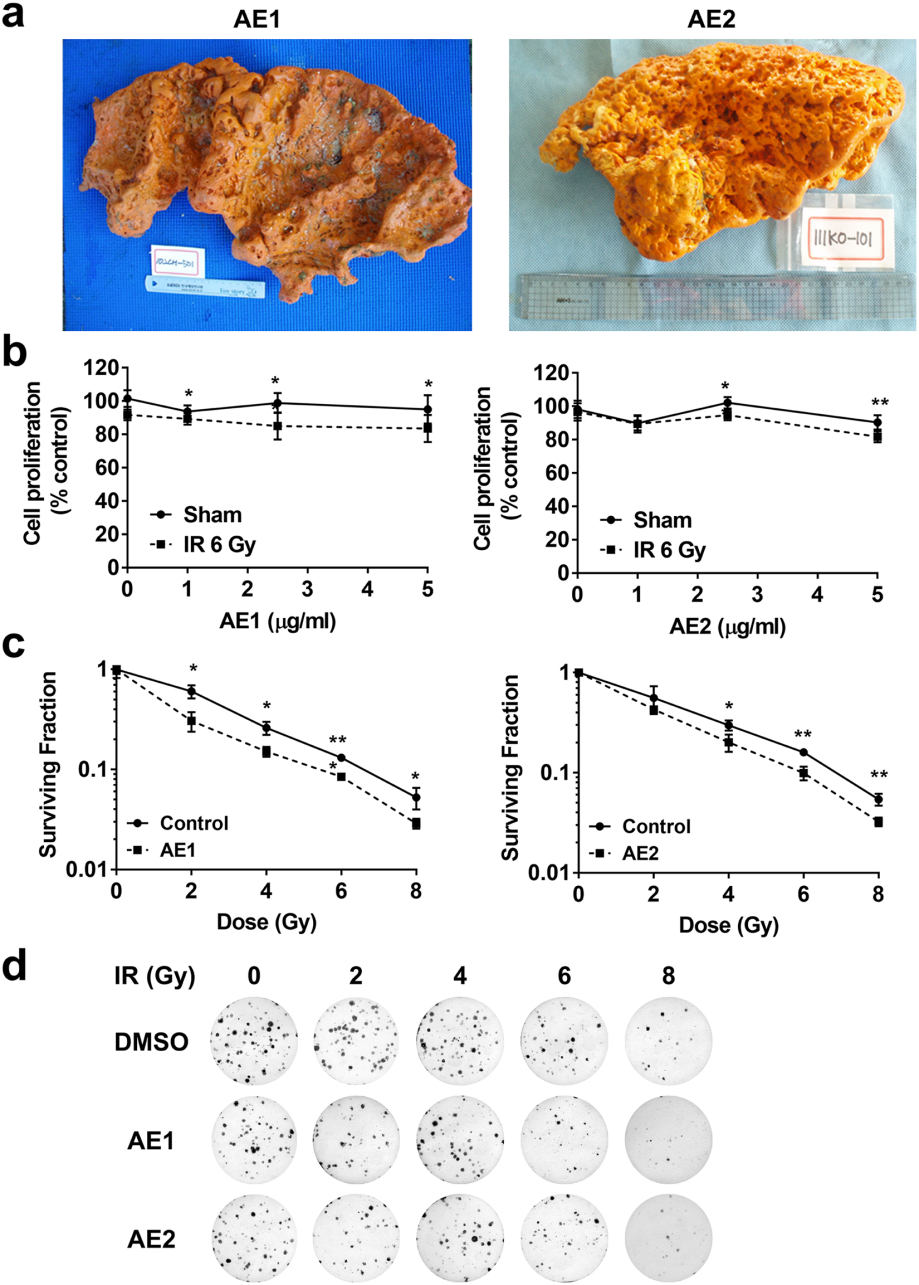
The *Agelas* extracts decrease clonogenic survival after IR treatment in human hepatocellular carcinoma cells. (**a**) Photographs of the original *Agelas* sp. specimens collected from Chuuk State and Kosrae States from which AE1 and AE2 samples were extracted, respectively. (**b**) AE1 and AE2 increased cytotoxic effect when combined with 6 Gy of IR. Data are presented as mean ± SD (n=7). *p < 0.05, **p < 0.01. (**c**) Clonogenic survival assay showed AE1 and AE2 but not #5 significantly increased radiation sensitivity of Hep3B cells. Data are presented as mean ± SD (n=3). *p < 0.05, **p < 0.01. (**d**) Photos of Hep3B colonies survived after exposure of the indicated doses of X-rays. Hep3B cells were pre-treated with 1 µg/mL of AE1 or AE2 for 3 h, followed by X-ray irradiations.

We screened radiosensitizing activity in three sponge extracts (#1, #2 and #5) which exhibited mild cytotoxicity. Two *Agelas* extracts, named AE1 (#1) and AE2 (#2) were prepared from the specimens collected from the oceans of Chuuk and Kosrae States, respectively, in Micronesia (Fig. 2a). A cytotoxicity assay showed that AE1 and AE2 further decreased cell proliferation at 5 µg/mL when combined with 6 Gy of IR (p < 0.05 and p < 0.01, respectively; Fig. 2b). We performed a clonogenic survival assay, the gold standard method for assessing radiation sensitivity, to confirm the radiosensitizing activity of the *Agelas* extracts. The Hep3B cells were pre-treated with 1 µg/mL of AE1 or AE2 for 3 h, followed by exposure to various doses of IR. After 1–2 weeks, the surviving colonies were stained and counted. Dose response curves showed that the pre-treatment with either AE1 or AE2 significantly decreased clonogenic survival after exposure to IR (Fig. 2c and d). In contrast, *Coscinoderma* sp. extract (#5) showed no radiosensitizing activity in Hep3B cells (Supplementary Fig. S1a,b). As a positive control, treatment with suberoylanilide hydroxamic acid (SAHA), a well-known radiosensitizer^16,17^ led to enhancement of radiation-mediated inhibition of proliferation and clonogenic survival of Hep3B cells (Supplementary Fig. S1c,d). Therefore, these data suggest that the *Agelas* extracts may contain bioactive molecules that enhanced the radiation effect.

### The *Agelas* extracts enhance radiation-induced apoptosis

**Figure 3:**
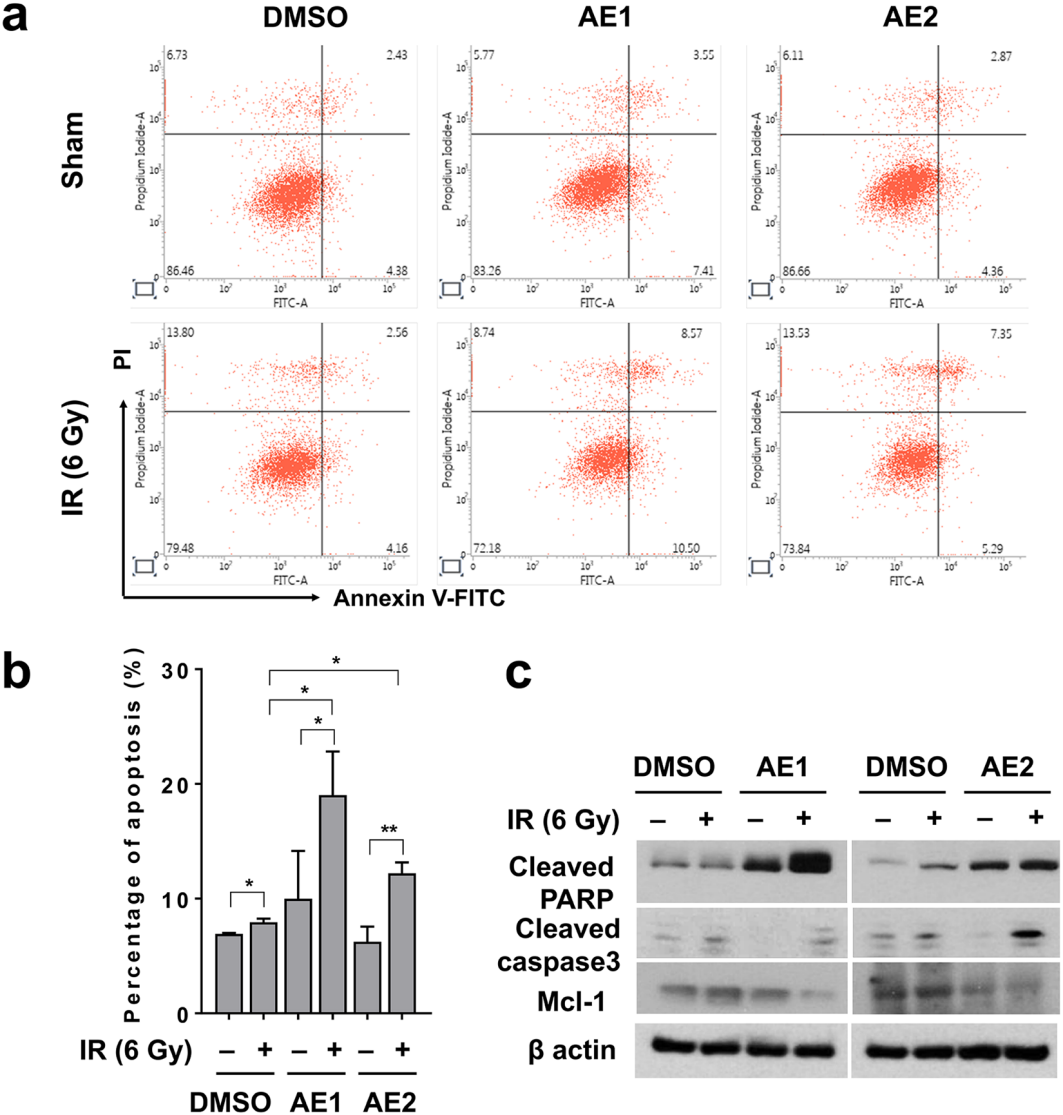
The *Agelas* extracts enhance IR-induced apoptosis. (**a**) Apoptosis was assessed by flow cytometry with FITC-conjugated annexin V/PI staining. Hep3B cells were pre-treated with 10 µg/mL of AE1 or AE2 for 3 h, followed by exposure to 6 Gy of IR. After 24 h of incubation, Hep3B cells were co-stained with annexin V-FITC and PI and was subjected to flow cytometer. (**b**) Quantification of apoptotic cell percentage showed the *Agelas* extracts enhanced IR-induced apoptosis. The data represent the means ± SD of three independent experiments (n=3; *p < 0.05, **p < 0.01). **(c)** Western blot analysis confirmed enhanced apoptosis by the *Agelas* extracts. Hep3B cell lysates were immunoblotted for the detection of cleaved PARP1, and Mcl-1. β-actin was used as a loading control. Uncropped images are shown in Supplementary Fig. S3.

We determined the effects of the *Agelas* extracts on apoptosis, a mode of radiation-induced death, to determine the mechanism of increased radiation sensitization. Flow cytometry analysis with annexin V staining showed that AE1 and AE2 did not change the percentage of apoptotic cells, and 6 Gy of IR induced apoptotic cell death (p < 0.05; Fig. 3a,b). The pre-treatment with either AE1 or AE2 significantly enhanced IR-induced apoptosis (p < 0.05; Fig. 3a,b). Western blot analysis further confirmed that the *Agelas* extracts increased apoptosis (Fig. 3c). IR-induced expression of cleaved PARP, a surrogate marker of intrinsic apoptosis signalling, were further increased by pre-treatment with AE1 or AE2. The anti-apoptotic protein, Mcl-1, was decreased by the pre-treatment. Thus, enhanced radiosensitization caused by the *Agelas* extracts may be attributed to increased apoptotic cell death.

### The *Agelas* extracts induce autophagy by stimulating endoplasmic reticulum stress

**Figure 4:**
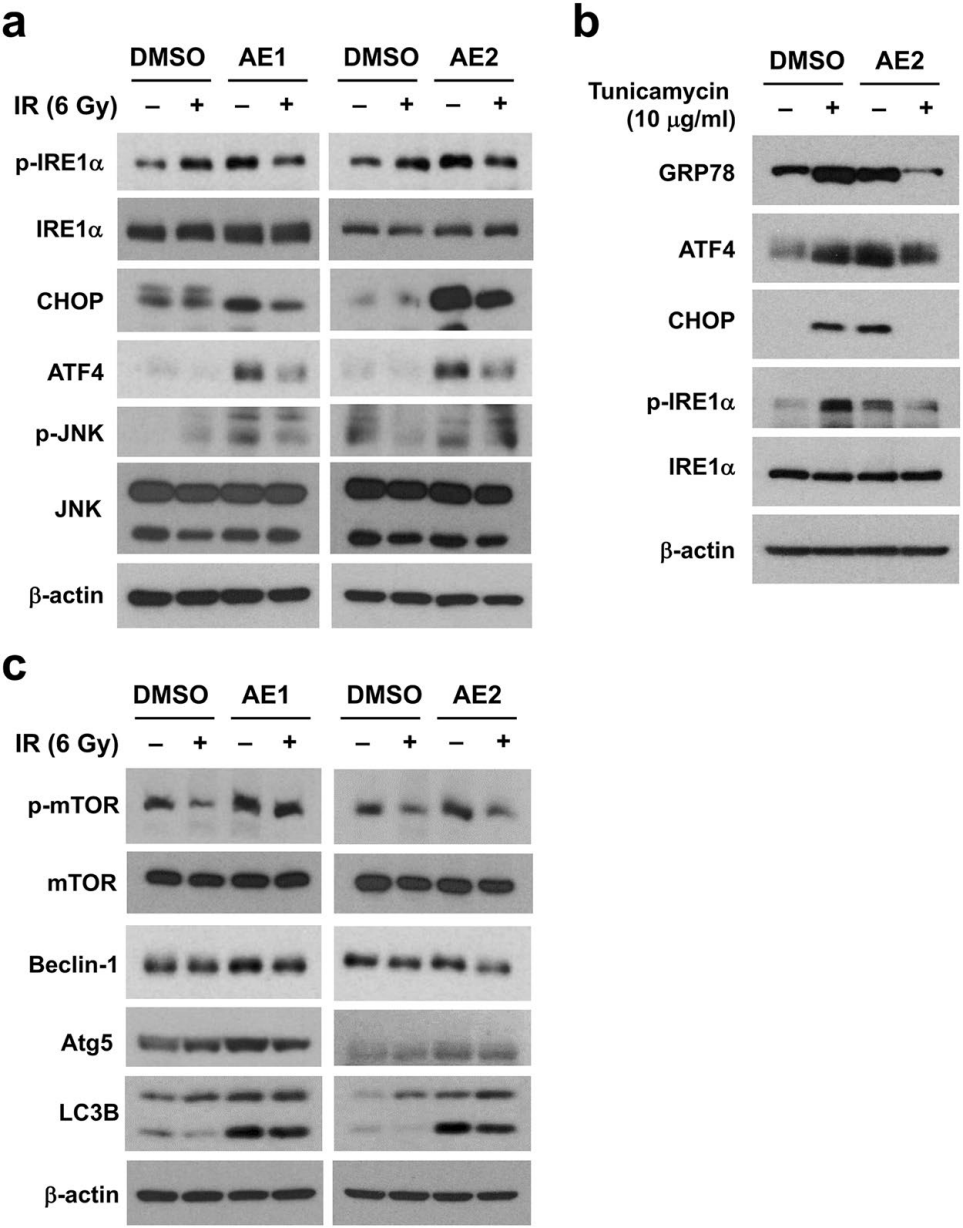
The* Agelas* extracts induce autophagy by stimulating ER stress. (**a**) Pre-treatment with the *Agelas* extracts led to induction of ER stress. Hep3B cells were pre-incubated with 10 µg/mL of AE1 or AE2 for 3 h, followed by 6 Gy of IR. At 24 h post-irradiation, the samples were prepared and subjected to Western blot analysis. Expression of unfolded protein response-related proteins, including phospho-IRE1α, total IRE1α, CHOP, ATF4, phospho-JNK and total JNK were upregulated by pre-treatment with AE1 and AE2 but diminished by co-treatment with 6 Gy of IR. β-actin was used as a loading control. (**b**) *A*g*elas* extract-induced ER stress signaling was suppressed by tunicamycin, a well-known unfold protein response inducer. Expression of GRP78, ATF4, CHOP, phospho-IRE1α was induced by either AE2 or tunicamycin, but was suppressed by the combination treatment. **(c)** Pre-treatment with the *Agelas* extracts led to induction of autophagy. Treatment with 10 µg/mL of AE1 or AE2 increased expression of autophagy-related proteins including phospho-mTOR, beclin-1, Atg5 and LC3B-II. Co-treatment with IR suppressed the *Agelas* extract-induced autophagy. Uncropped images are shown in Supplementary Fig. S3.

Autophagy induction is a survival mechanism activated by exposure to IR, so autophagy is considered a potential therapeutic target^18,19^. The effect of the *Agelas* extracts on endoplasmic reticulum (ER) stress, which triggers autophagy, was determined by protein expression of the unfolded protein response (UPR) pathways^20^. Major UPR pathways are comprised of UPR sensors such as PERK, IRE1α, and ATF6 with their downstream effectors. Both AE1 and AE2 activated the UPR pathways, as indicated by increased expression of phospho-IRE1α, CHOP, ATF4, and phospho-JNK (Fig. 4a). In contrast, *Coscinoderma* sp. extract (#5) did not induce ER stress (Supplementary Fig. S2a). IR alone dose-dependently increased phospho-IRE1α, GRP78 and ATF4 (Supplementary Fig. S2b). The addition of IR suppressed the *Agelas* extract-mediated UPR activation (Fig. 4a). Similarly, tunicamycin, a strong ER stress inducer^21^, suppressed expression of UPR proteins including GRP78, ATF4, CHOP and phospho-IRE1α when co-treated with AE2 (Fig. 4b). Next, we determined the effect of the *Agelas* extracts on autophagy since excessive ER stress induces autophagy. Both AE1 and AE2 induced the expression of the autophagy-related proteins including phospho-mTOR, beclin-1, Atg5, and LC3B-II in Hep3B cells (Fig. 4c). Combined treatment with 6 Gy of IR diminished the *Agelas* extract-induced autophagy (Fig. 4c), which was apparent in the AE2-treated samples. A similar pattern was observed in the autophagy and UPR responses, so the *Agelas* extracts may trigger autophagy signalling via ER stress.

### The *Agelas* extracts augment radiation-induced reactive oxygen species production

**Figure 5:**
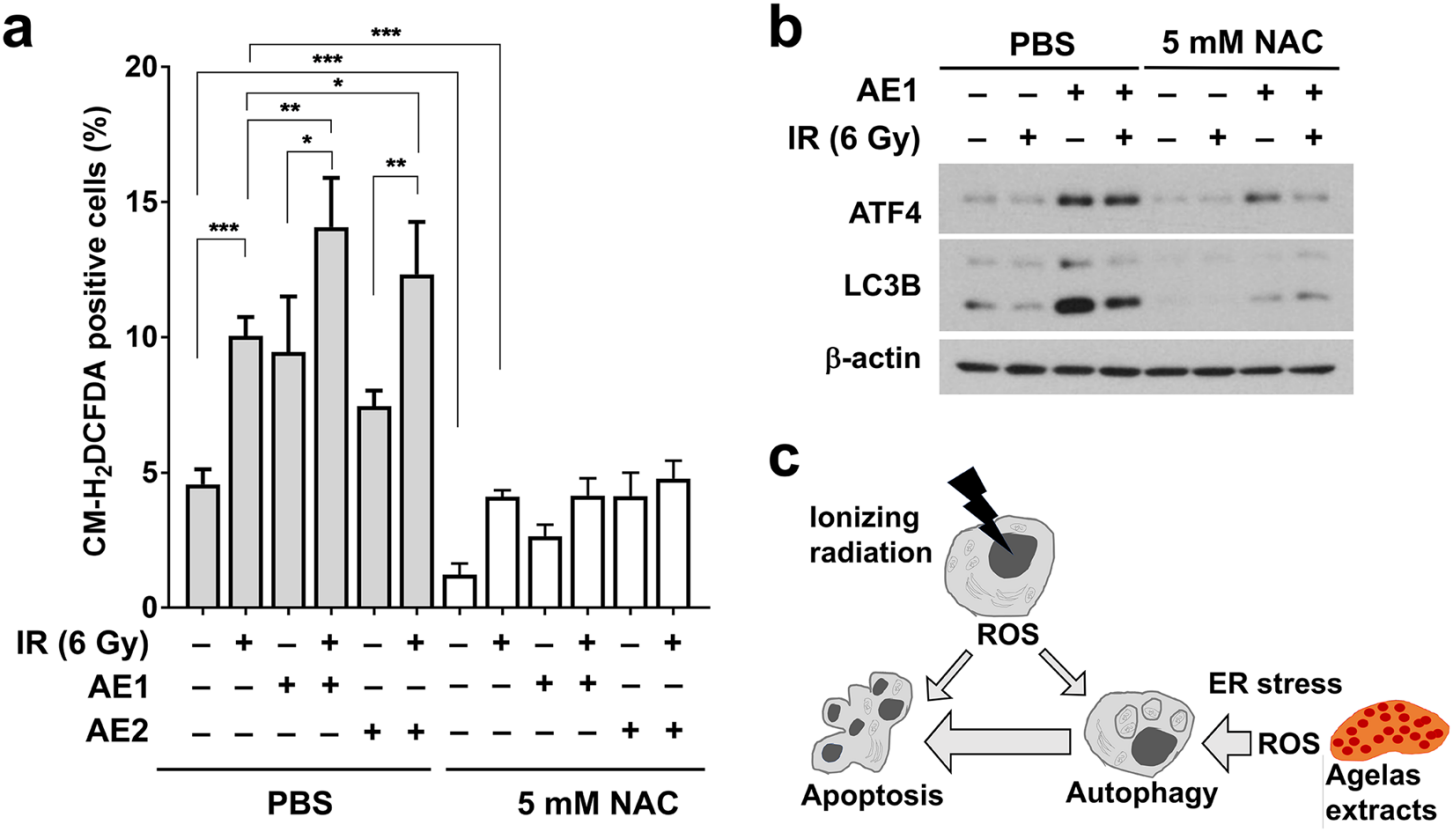
The *Agelas* extracts augment IR-induced ROS production. (**a**) Hep3B cells were pre-incubated with 10 µg/mL of AE1 or AE2 and CM-H_2_DCFDA for 3 h, followed by exposure to 6 Gy of IR. After 48 h of incubation, intracellular ROS levels were determined by DCF fluorescence using Flow cytometry. *p < 0.05, **p < 0.01, ***p < 0.001. (**b**) N-acetylcysteine (NAC) abolished the induction of ER-stress and autophagy by the *Agelas* extracts. Pre-treatment with 5 mM NAC decreased AE1-induced expression of ATF4 and LC3B-II. PBS, phosphate buffered saline. Uncropped images are shown in Supplementary Fig. S3. **(c)** Schematic diagram of how the *Agelas* extracts increase radiosensitization in Hep3B cells.

Reactive oxygen species (ROS) are a common source of IR-induced DNA damage and ER stress. We determined the effect of the *Agelas* extracts on ROS production by using the redox sensitive fluorescence dye, CM-H_2_DCFDA. Treatment with 6 Gy of IR significantly increased intracellular ROS levels, compared to the sham-treated control (p < 0.001, Fig. 5a). Pre-treatment with either AE1 or AE2 also induced intracellular ROS production and further augmented ROS levels when combined with IR (Fig. 5a). We determined the effect of N-acetylcysteine (NAC), a common ROS scavenger, on *Agelas* extract-mediated ER stress and autophagy. Pre-treatment with 5 mM NAC decreased intracellular ROS levels and abolished ROS augmentation by either IR or the *Agelas* extracts (p < 0.001, Fig. 5a). The NAC pre-treatment strongly suppressed the AE1-induced expression of ATF4 and LC3B-II (Fig. 5b). The suppressive effect of NAC was also observed with AE2 (data not shown). These results suggest that the increased ROS levels from *Agelas* extract treatment may induce ER stress and autophagy, leading to the radiosensitization effect (Fig. 5c).

## Discussion

Due to highly diverse marine habitats, metabolites isolated from marine organisms are considered a rich resource of bioactive natural products. Numerous efforts to screen bioactive molecules have resulted in identification of various metabolites that possess potential anti-cancer activity^2,5,22^. Several marine-derived anti-cancer drugs are already FDA-approved or being tested in clinical trials^2,4^. For example, cytarabine, a synthetic pyrimidine nucleoside originally isolated from the Caribbean sponge *Tethya crypta* has been approved for leukaemia. Trabectedin is an FDA-approved marine metabolite of *Ecteinascidia turbinata*, a tunicate from the Caribbean and Mediterranean Sea, for soft tissue sarcoma treatment^2,4^. While the list of marine-derived drugs tested in clinical trials is rapidly growing, none have been tested as radiation therapy adjuvants.

HCC is a leading cause of cancer-related mortality because patients with HCC are often diagnosed at advanced stages where curative approaches are not feasible. Radiation therapy is an effective option in this situation and sensitizers that improve the efficacy of radiation therapy have been investigated^9,15^. We obtained extracts of marine sponges collected from the deep sea near Micronesia and screened their radiosensitizing activity. These novel marine-derived extracts could have potentially applications with radiation therapy for HCC treatment.

Initial blind tests were performed with six marine-derived extracts based on a cell proliferation assay. Among them, two samples slightly increased radiation-mediated cytotoxicity, which were obtained from *Agelas* sp. separately collected from oceans near the Chuuk and Kosrae states. The radiosensitizing effect of the *Agelas* extracts was confirmed by clonogenic survival assay, the gold standard method for measuring radiation sensitivity. The *Agelas* extracts alone did not demonstrate cytotoxicity, but extracts from *Theonella* sp. and *Haliclona* sp. had strong cytotoxic effects on HCC cells. *Coscinoderma* extract showed the similar cytotoxic profile with *Agelas* extracts but no radiosensitizing activity, indicating a distinctive feature of *Agelas* extracts from others.

We investigated the effects of *Agelas* extracts on apoptosis and autophagy to elucidate the underlying mechanisms of enhanced radiation-mediated cytotoxicity. Flow cytometry and biochemical analyses revealed that neither extract increased apoptotic death in Hep3B cells. Apoptosis was enhanced when the extracts were combined with IR, suggesting that radiosensitization may be related to increased apoptotic death. IR activates survival signalling pathways, and autophagy is a cellular mechanism that confers radioresistance^18,19,23,24^. Preclinical studies have shown that inhibiting autophagy via siRNA against beclin 1 and ATG5, or pharmacological inhibitors such as 3-MA, increase the efficacy of radiotherapy in breast cancer, glioma, colorectal, and oesophageal cancer cells^25–27^. This suggests that autophagy may be a potential therapeutic target in cancer patients that receive radiation therapy. Autophagy induction via AKT or mTOR inhibitors also leads to radiosensitization in lung cancer and glioma^28,29^. Since autophagy is implicated in cancer cell survival and death, treatment strategies must be carefully designed. We found that the *Agelas* extracts alone greatly induced autophagy, but they suppressed autophagy and induced apoptosis when they were combined with IR.

Induction of ER stress and UPR activation stimulated autophagy, which is implicated in cancer progression and therapeutic response. IR induces the unfolded protein response and ER stress in this study and others^18,19^. *Agelas* extracts dramatically induced ER stress, but *Coscinoderma* extract did not, suggesting that the excessive ER stress may be closely related to the radiosensitization effect. Moreover, combination of *Agelas* extracts with tunicamycin, a well-known ER stress inducer, suppressed UPR activation as seen in their combination with IR. The ROS accumulated by water radiolysis during radiation treatment is a source of DNA oxidative damage and a critical inducer of ER stress. Both *Agelas* extracts increased expression of the proteins related to ER stress and autophagy as well as ROS production. Co-treatment with the ROS scavenger, NAC, suppressed the *Agelas* extract-mediated autophagy induction. This result suggested that ROS production by the *Agelas* extracts may induce ER stress and autophagy. Taken together, our data suggest that combination of the *Agelas* extracts with IR may lead to excessive ROS production, thereby shifting the balance from survival to death (Fig. 5c).

Sponges are a primitive marine invertebrate that produce over 5,300 different metabolites, including alkaloids, glycosides, macrolides, and terpenoids. Sponges have been investigated and utilized as a rich source of natural products for anti-cancer drug discovery^3,6^. *Agelas* sp. contain several well-characterized metabolites, such as agelasines and oroidin. A variety of structural modifications have been attempted to increase the efficacy and specificity of their bioactivity^30,31^. To our knowledge, this is the first report describing the radiosensitizing activity of marine sponge extracts and providing mechanistic insights enhanced radiation efficacy by *Agelas* extracts. Further studies are warranted to identify and develop natural radiosensitizers from the marine sponges *Agelas* extracts and evaluate *in vivo* efficacy in animal models.

## Materials and Methods

### Preparation of marine sponge extracts

The *Agelas* sponges were collected by hand using SCUBA at a 10-meter depth off the shores of Chuuk and Kosrae, the Federated States of Micronesia. The lyophilized sponge was extracted with MeOH and CH_2_Cl_2_ at room temperature for 12 h. The combined extract was partitioned between *n*-BuOH and H_2_O to remove salts, and the organic layer was concentrated under vacuum and dissolved in DMSO for the evaluation of biological activity.

### Cell culture

Human hepatocellular carcinoma Hep3B, human breast adenocarcinoma MDA-MB-231, human colorectal adenocarcinoma HT29 cells were purchased from the Korean Cell Line Bank (Seoul National University, Seoul, Korea) or from the American Type Culture Collection (ATCC, Manassas, VA, USA). Hep3B and MDA-MB-231 cells were grown in Dulbecco’s modified Eagle’s medium (DMEM) supplemented with 10% foetal bovine serum (FBS), 1% Antibiotic-Antimycotic (AA, Thermo Fisher Scientific, Waltham, MA, USA), and 25 mM HEPES (Gibco, Carlsbad, CA, USA). HT29 cells were grown in RPMI 1640 medium supplemented with 10% FBS, 1% AA. Cells were maintained in a humidified 37 °C incubator with 5% CO_2_.

### Cell viability assay

Cell viability was determined by Cell Counting Kit-8 assay (CCK-8, Dojindo Laboratories, Kumamoto, Japan). Hep3B cells were seeded at 1 × 10^3^ cells/well into a 96-well plate and incubated with various concentrations of the extract samples for 24, 48, and 72 h. After 2 h of incubation with CCK-8 solution, the absorbance at 450 nm was determined using a SpectraMax i3 microplate reader (Molecular Devices, Sunnyvale, CA, USA). The relative cell viability was calculated as percentage of untreated control.

### Irradiation experiments

X-ray irradiation was performed as previously described^32^. Briefly, Hep3B cells were irradiated with 6-MV X-rays at a dose rate of 3.96 Gy per min using a linear accelerator Varian Clinac 6EX machine (Varian Medical Systems, Palo Alto, CA, USA). The cell dishes were placed under 2 cm thickness solid water phantom with source surface distance of 100 cm and field size of 30 × 30 cm. The X-ray absolute dose was calibrated according to TG-51 and verified with Gafchromic film to 1% accuracy.

### Clonogenic survival assay

Clonogenic assay was performed as previously described^32,33^. Hep3B cells were seeded into a six well plate in triplicate and then incubated overnight (200 cells for 0 Gy and 2 Gy; 400 cells for 4 Gy; 800 cells for 6 Gy; 1000 cells for 8 Gy). The cells were pre-treated with 5 µg/mL of sponge extracts or 1 μM suberoylanilide hydroxamic acid (SAHA; Sigma, St. Louse, MO, USA) for 3 h and were subsequently irradiated with increasing doses of 0, 2, 4, 6, and 8 Gy of IR. After incubation for 7–14 days, surviving cells were fixed and stained with 1% crystal violet and the colonies containing 50 or more cells were manually counted. Plating efficiency was calculated as % of colonies from seeded cells and cell survival fraction at each irradiation dose was determined by dividing the plating efficiency of the irradiated cells by that of the sham-treated control. The survival curves were drawn using GraphPad Prism 7.02 (GraphPad Software, La Jolla, CA, USA).

### Apoptosis assay

Apoptosis was assessed by flow cytometry after annexin V/propidium iodide (PI) staining. 1 × 10^5^ Hep3B cells were plated in 6-well plates and next day the cells were pre-treated with 10 µg/mL of AE1 or AE2 for 3 h, followed by exposure to 6 Gy of IR. After 72 h incubation, cells were treated with trypsin, washed with PBS (pH 7.4), and stained with annexin V-FITC (BD Pharmingen, San Diego, CA, USA) and 2 μg/mL PI in annexin V binding buffer (10 mM HEPES, pH 7.4, 140 mM NaCl, 2.5 mM CaCl_2_) for 15 min at 37 °C in the dark. Samples were analyzed by using a BD FACSVerse flow cytometer (Bectron-Dickinson, CA, USA) and data acquisition software, BD FACSuite.

### ROS measurement

Hep3B cells were pre-treated with 5 mM N-acetylcysteine for 1 h before the incubation with the marine sponge extracts. Cells were pre-incubated with 10 µg/mL of AE1 or AE2 and 2 μM Carboxy-H_2_DCFDA for 3 h. Intracellular ROS level was analyzed by flow cytometry 48 h post-irradiation (FACSVerse, Becton-Dickinson, CA, USA).

### Western blot analysis

Hep3B cells were lysed in a lysis buffer (20 mM Tris, pH 8.0) containing 137 mM NaCl, 10% glycerol, 1% nonidet P-40, 10 mM EDTA, 100 mM NaF, 1 mM phenylmethylsulfonyl fluoride, and 10 mg/mL leupeptin. The supernatant was collected after centrifugation at 13,000 rpm for 15 min. Protein concentration in each lysate was determined using Bio-Rad protein assay reagent (Bio-Rad, Richmond, CA, USA). Equal amounts of proteins were separated by SDS-PAGE and transferred to nitrocellulose membranes (Bio-Rad). Blots were blocked overnight with 5% skim milk in PBS at 4 °C, followed by probing with primary antibodies overnight. After incubation with secondary antibodies for 1 h, bands of interest were visualized with Amersham enhanced chemiluminescence detection reagents (GE healthcare, Piscataway, NJ, USA).

### Statistical analysis

Data are presented as mean ± standard deviation (SD). Statistical analysis was performed using GraphPad Prism 7.02. Statistical significance of differences between experimental groups was calculated with an unpaired, two-tailed, Student’s t-test. P values < 0.05 were considered statistically significant.

## Electronic supplementary material

Supplementary figures
