# Supplementary material for: Radiosensitization by Marine Sponge Agelas sp. Extracts in Hepatocellular Carcinoma Cells with Autophagy Induction
Source: Sci Rep. 2018 Apr 20;8:6317. doi: 10.1038/s41598-018-24745-w (PMC5910397; doi:10.1038/s41598-018-24745-w)
Supplement: Supplementary file 1 — Supplementary figures [file 41598_2018_24745_MOESM1_ESM.pdf]

# Supplementary Figures

## Radiosensitization by Marine Sponge *Agelas* sp. Extracts in Hepatocellular Carcinoma cells with Autophagy Induction

**Changhoon Choi[1], Arang Son[1], Hyi-Seung Lee[2], Yeon-Ju Lee[2] and Hee Chul Park[1,3]**

[1]Department of Radiation Oncology, Samsung Medical Center, Seoul, 06351, South Korea,

[2]Marine Natural Product Chemistry Laboratory, Korea Institute of Ocean Science, and Technology, Ansan 426-744, South Korea

[3]Sungkyunkwan University School of Medicine, Seoul, 06351, South Korea

Correspondence and requests for materials should be addressed to H.C.P. (E-mail: hee.ro.park@samsung.com)

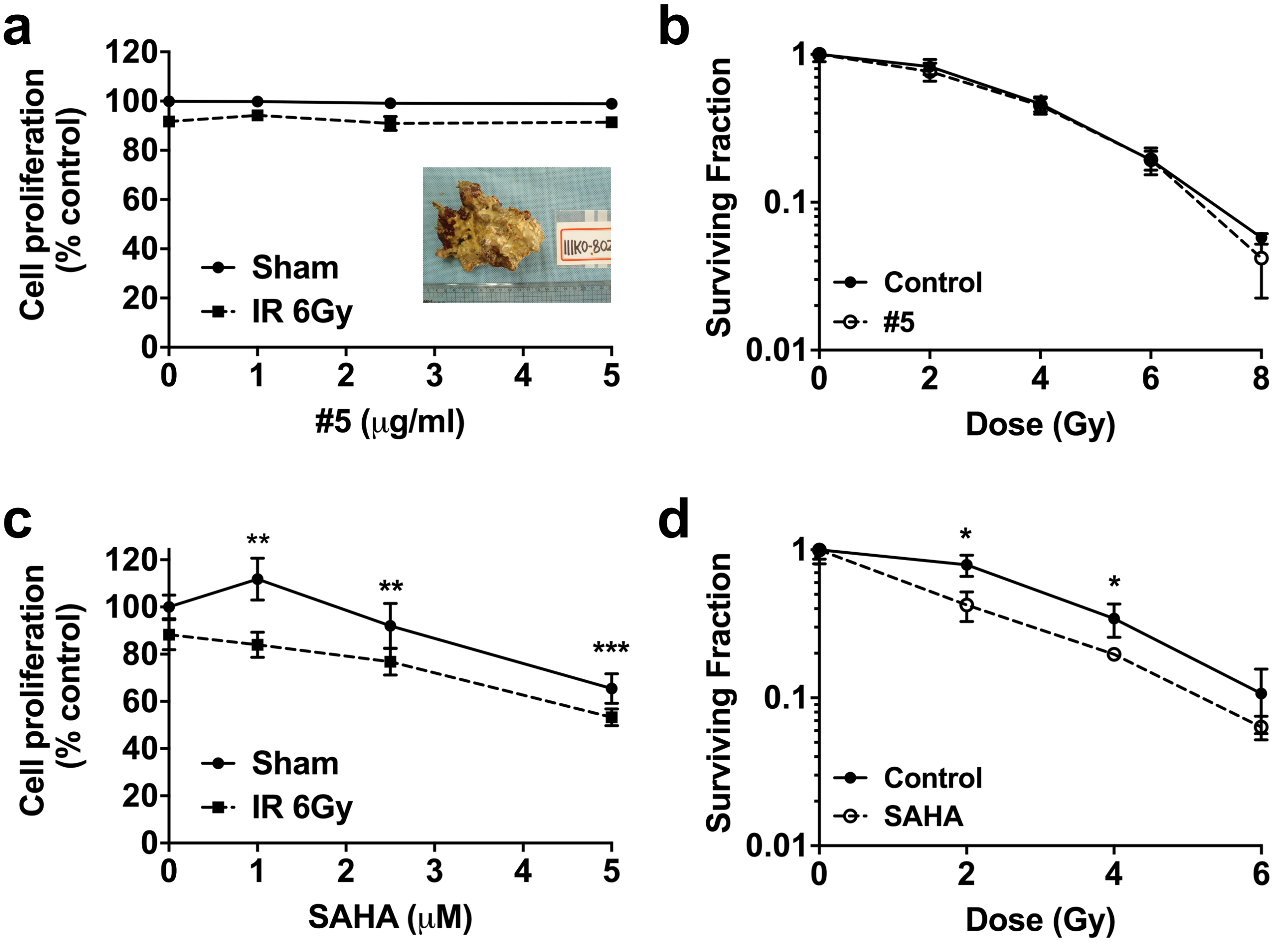


**Supplementary Fig. S1. Effect of Coscinoderma extract (#5) and SAHA on radiosensitivity of Hep3B cells.** When combined with 6 Gy of IR, extract from Coscinoderma sp. (see a photo) showed no additive effect on cell proliferation **(a)** and clonogenic survival **(b)**. In contrast, SAHA enhanced radiation effect on cell proliferation **(c)** and clonogenic survival **(d)**. Data are mean ± S.D.; $^{*}p<0.05$;$^{**}p<0.01$;$^{***}p<0.001$.

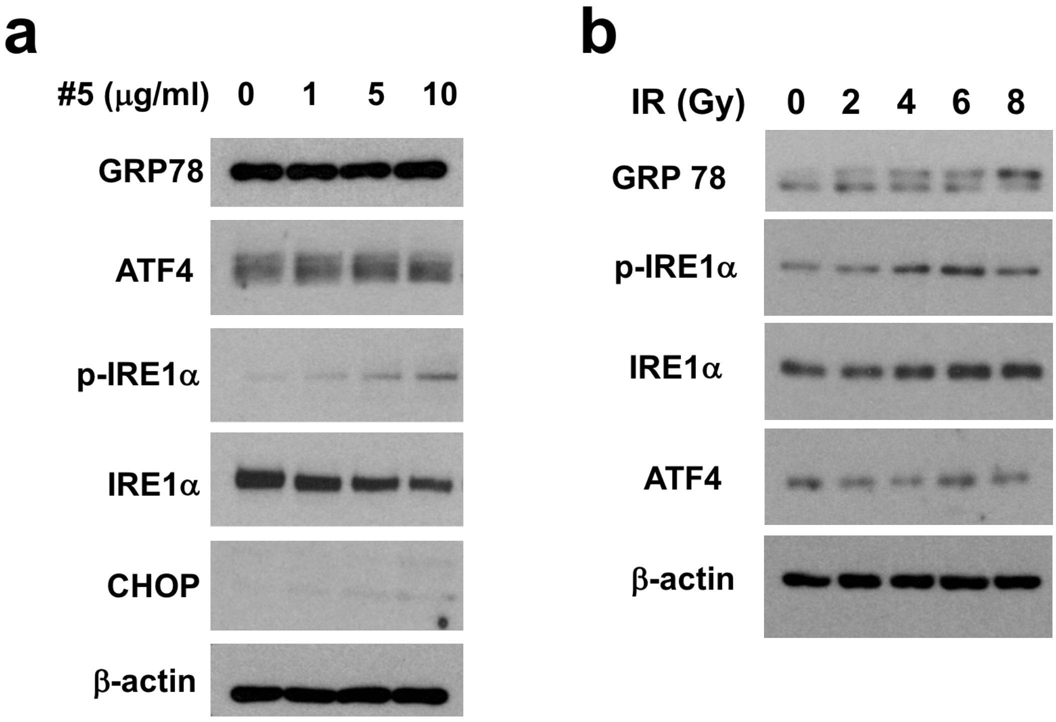


**Supplementary Fig. S2. Effect of Coscinoderma extract (#5) and IR on ER stress in Hep3B cells. (a)** Hep3B cells were treated with the indicated amounts of Coscinoderma extract and were harvested after 24 h of incubation. Western blot analysis showed little increase in expression of ER stress-related proteins. **(b)** After 24 h of exposure to the indicated dose of X-rays, Hep3B cells were subjected to Western blot analysis. IR increased ER stress-related proteins dose-dependently. Uncropped images are shown in Supplementary Fig. S3.

# Full-length blots used in Fig. 3c

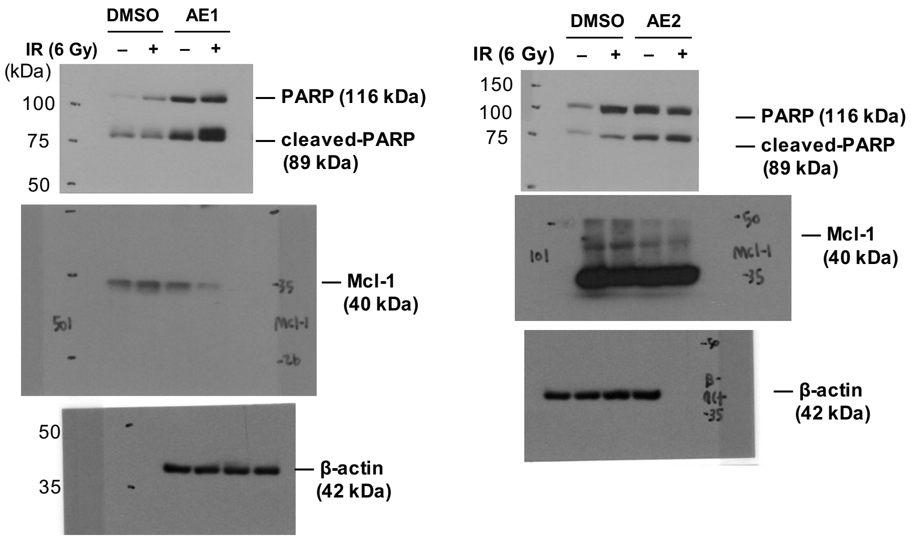


# Full-length blots used in Fig. 4a

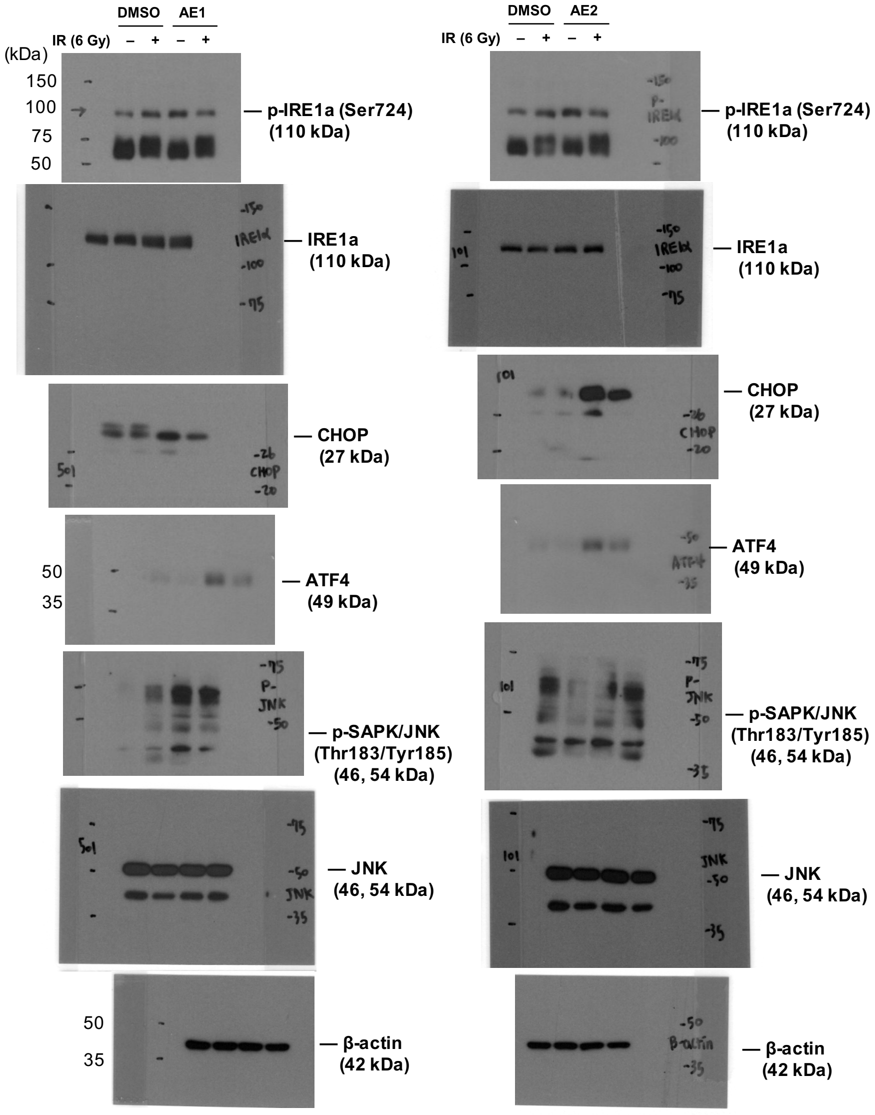


**Supplementary Fig. S3. Uncropped images of western blots.**

## Full-length blots used in Fig. 4b and 4c

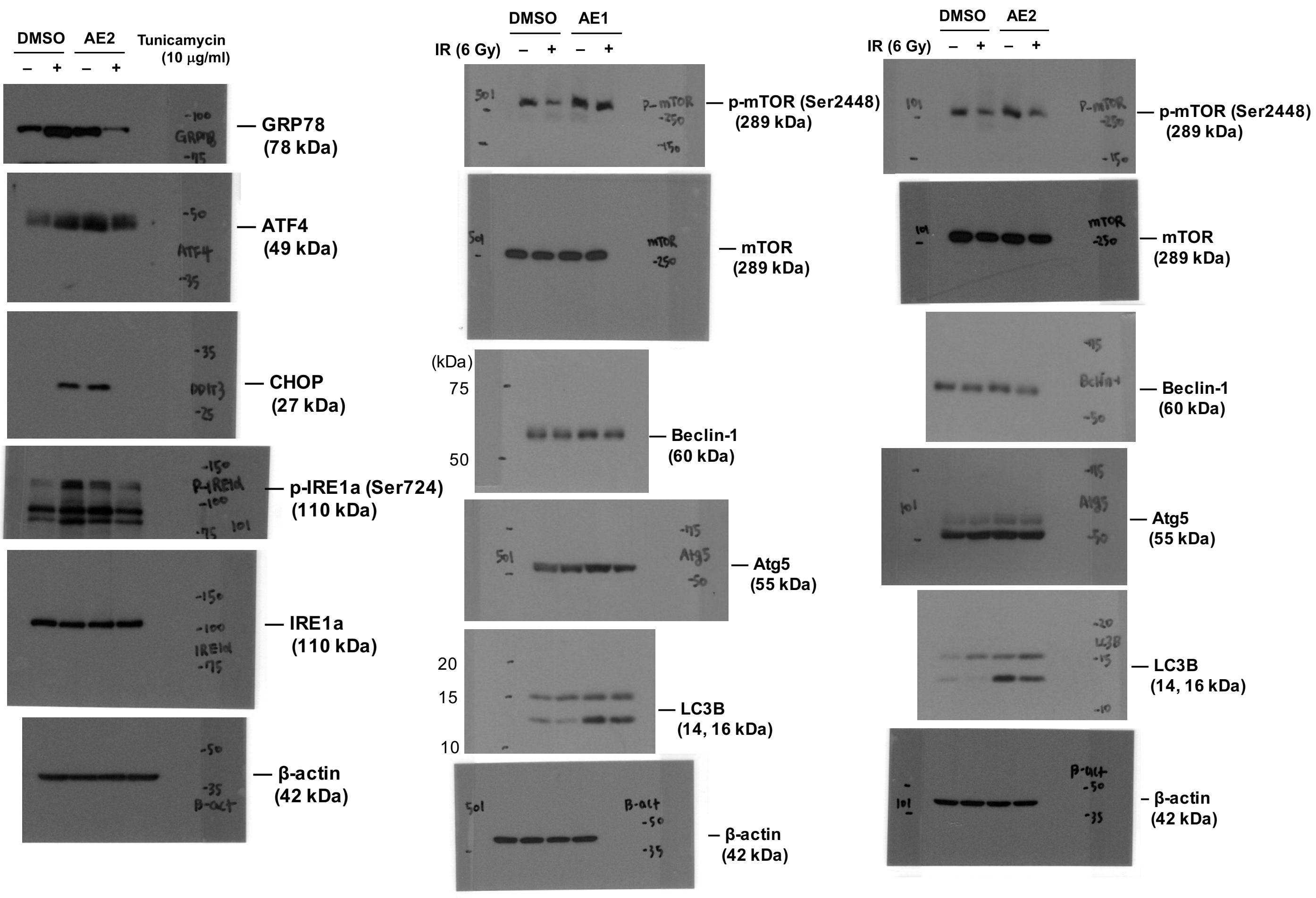


## Full-length blots used in Fig. 5b

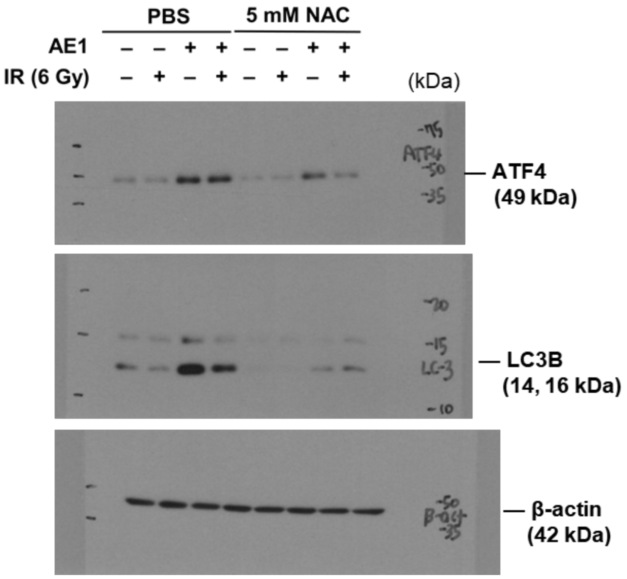


**Supplementary Fig. S3. Uncropped images of western blots.**

# Full-length blots used in Supplementary Fig. S2a and S2b

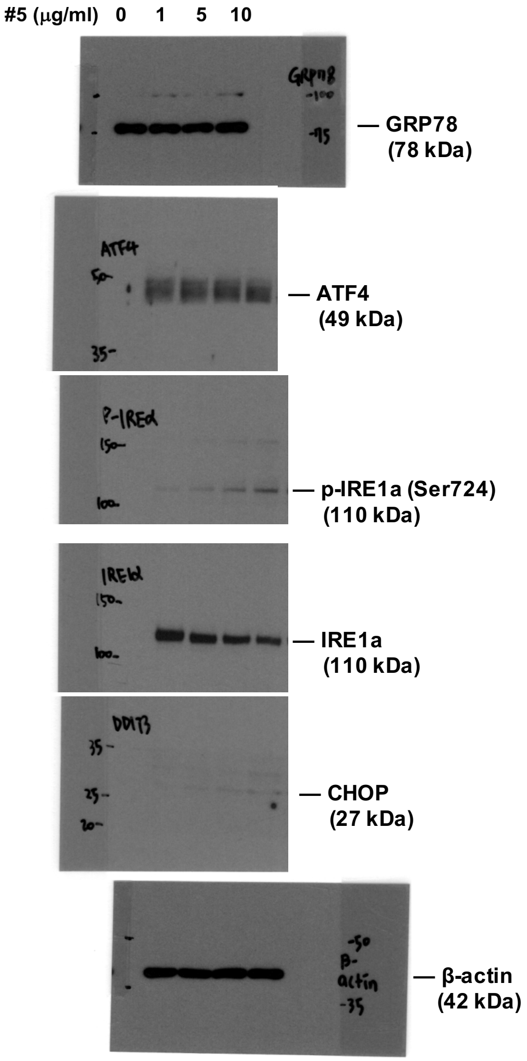


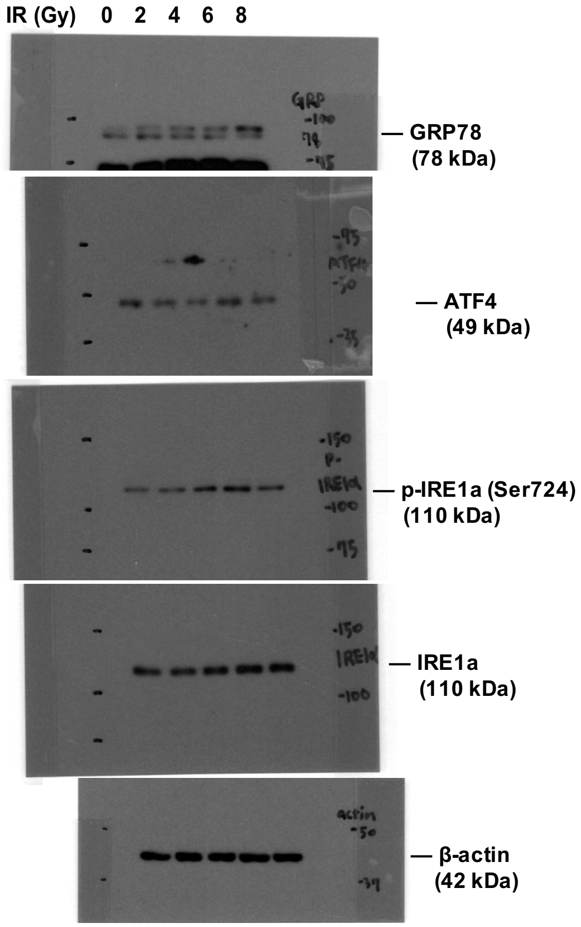


**Supplementary Fig. S3. Uncropped images of western blots.**
